# Breaking boundaries in subduction science

**DOI:** 10.1038/s41467-020-19930-3

**Published:** 2020-11-25

## Abstract

Subduction is the primary driver of plate tectonics, yet we still do not fully understand how subduction zones initiate or the budgets of life-supporting elements recycled via subduction. At *Nature Communications*, we advocate for more transdisciplinary initiatives and collaborative projects, which are essential if we are to continue to bring new dynamics to subduction research.

Subduction zones pose threats to many societies, as they are the locus of destructive volcanic eruptions, earthquakes, and tsunamis. Earth’s rocky outer layer is continuously being recycled into the mantle via subduction, where one tectonic plate descends into the mantle beneath another plate. Water trapped in the subducting plate is released into the mantle at depth, which in turn enhances mantle melting, leading to the development of volcanoes on the overriding plate. In addition to water, essential nutrients such as carbon and sulfur are also carried down into the mantle at subduction zones, and are released back into the atmosphere via volcanic eruptions^1^. Subduction has therefore not only caused destruction, but also provided a critical exchange of life-supporting elements between the biosphere and geosphere over Earth’s history.

Only through collaborating and communicating between the disparate fields of geophysics, geodynamics, geodesy, geochemistry, petrology and biogeochemistry can we continue to make significant progress in understanding one of the key driving forces of our planet.

Despite their importance, subduction zones are only a relatively recent scientific discovery. But today, plate movements are monitored on an almost daily basis, and features of the Earth’s mantle are mapped at unprecedented detail. A collection in *Nature Communications* showcases a selection of recent significant advances in subduction science, highlighting studies which go beyond traditional disciplinary boundaries to answer fundamental questions regarding the workings of our planet.

**Figure:**
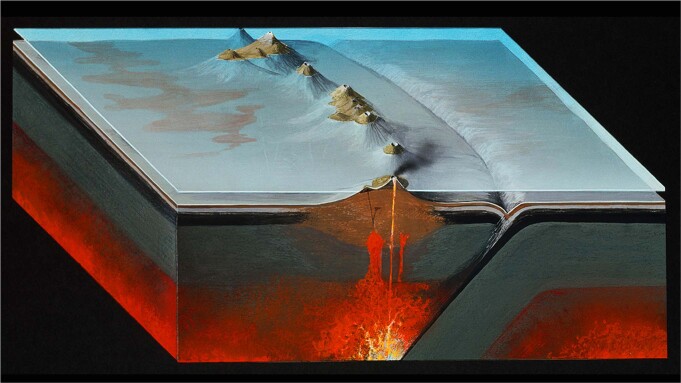
Natural History Museum, London/Alamy

The initiation of the first subduction zones on the early Earth likely had major implications for carbon recycling, with consequences for the rise of atmospheric oxygen and thus the development of complex life^2^. But when and how tectonic plates first descended into the mantle to create self-sustaining subduction zones remains one of the major outstanding questions in Earth science. This difficulty in defining of the onset of plate tectonics is in part due to both the sparse record of early Earth rocks and the ambiguity in our understanding of the dynamics of early subduction. As a consequence, estimates for the onset of plate tectonics still range from ~4 to ~0.8 billion years ago^3^. Needless to say, narrowing down this time window should remain a research priority. With such sparse data from the early Earth it can be all too easy to fall in the trap of hypothesis-fitting or pre-conditioned ideas. Therefore, only by rigorously combining multidisciplinary lines of evidence with open minds can the community continue to progress.

In addition to the complexities of understanding the onset of plate tectonics billions of years ago, it is still unknown how geologically recent subduction zones initiate. We know that new subduction zones must form to maintain plate tectonics, yet there are no present-day examples of ongoing subduction zone initiation and the formation of new subduction zones leaves very few process-specific geologic traces^4^. Numerical geodynamic simulations of subduction zone initiation have sparked an exciting ongoing debate over the forces required for a plate to start descending into the mantle. However, the results from any numerical simulation are reliant on the strength of the input parameters, and therefore there is an urgent need for further geological constraints on subduction zone initiation. The Izu-Bonin-Mariana subduction zone is the most complete magmatic record of subduction zone initiation to date. As a result, it has been the site of significant intensive study by geochemists and petrologists, and is often used to constrain geodynamic models. The Izu-Bonin-Mariana system is therefore rightly the type-location of subduction zone initiation, however, it is likely that the tectonic history, force balance and magmatic products of this event were unique, and may not be applicable to all initiation events^4,5^.

A recent collaborative project characterised thirteen subduction initiation events over the last 100 million years, by combining evidence from geophysical imaging and geochemical records with results from plate tectonic reconstructions and geodynamic models^4^. However, the geological constraints on many of these young subduction events are sparse and need to be filled imminently by the community, requiring careful communication between various research groups. Filling in such data gaps could lead to exciting new advances in understanding the variations between different subduction zone initiation events, bringing new insights to our understanding of plate tectonics. Only through efficiently collaborating and communicating between the disparate fields of geophysics, geodynamics, geodesy, geochemistry, petrology and biogeochemistry can we continue to make significant progress in understanding one of the key driving forces of our planet.
